# Chronic Rhinosinusitis: Does Allergy Play a Role?

**DOI:** 10.3390/medsci7020030

**Published:** 2019-02-18

**Authors:** Sonya Marcus, John M. DelGaudio, Lauren T. Roland, Sarah K. Wise

**Affiliations:** Department of Otolaryngology-Head and Neck Surgery, Emory University, Atlanta, GA 30308, USA; sonya.marcus@emory.edu (S.M.); jdelgau@emory.edu (J.M.D.); lauren.roland@emory.edu (L.T.R)

**Keywords:** allergy, chronic rhinosinusitis, polyposis, allergic fungal rhinosinusitis, central compartment atopic disease

## Abstract

A few chronic rhinosinusitis (CRS) variants have demonstrated a strong association with environmental allergy, including allergic fungal rhinosinusitis (AFRS) and central compartment atopic disease (CCAD). However, the overall relationship between CRS and allergy remains poorly defined. The goal of this review is to evaluate the relationship between CRS and allergy with a focus on specific CRS variants.

## 1. Introduction 

Chronic rhinosinusitis (CRS) is an inflammatory condition within the paranasal sinuses that persists for more than 12 weeks [1]. It affects approximately 5% of the US population, with an estimated overall direct cost burden of about US $8.6 billion per year [2]. Given this substantial societal burden, significant research has been dedicated to better understanding CRS. 

In recent decades, we have gained an increased understanding that CRS is not a uniform disease process but rather encompasses several different phenotypes and endotypes [3]. Historically, CRS has been divided into two main phenotypes: CRS with nasal polyposis (CRSwNP) and CRS without nasal polyposis (CRSsNP). However, within these broad phenotypes, various endotypes exist. For example, within the CRSwNP patient population, allergic fungal rhinosinusitis (AFRS) and aspirin exacerbated respiratory disease (AERD) represent clinically distinct entities requiring differing management. 

Furthermore, CRS is a multifactorial disease process with genetic, environmental, bacterial and immunologic contributions, among other etiologies. In addition, allergic diseases, especially immunoglobulin E (IgE)-mediated inflammatory processes such as allergic rhinitis (AR) may influence the development and progression of CRS and should be considered in the CRS work-up and management [4]. However, the role of allergy as a comorbidity for CRS remains incompletely understood and appears to have a greater association with certain CRS endotypes. 

Therefore, the goal of this review is to examine the relationship between allergy and CRS, with a focus on specific CRS endotypes.

## 2. Association between Allergic Rhinitis and Chronic Rhinosinusitis: Overview

There are several proposed mechanisms by which allergy may contribute to CRS. Yet, despite evidence regarding a pathophysiologic association, clinical studies demonstrating a relationship between allergy and CRS have been conflicting [5], especially when patients are subtyped by broad phenotypic categories (CRSwNP and CRSsNP).

There is, however, strong evidence for an association with certain CRS subtypes, including allergic fungal rhinosinusitis (AFRS) and the more recently described central compartment atopic disease (CCAD), which will be individually discussed within this review.

## 3. Association between Allergic Rhinitis and Chronic Rhinosinusitis: Pathophysiology

Significant study has been dedicated toward better understanding the inflammatory profiles that contribute to the development and propagation of CRS. Broadly, CRS has been divided into CRSwNP and CRSsNP, but further classification is based upon association with a type 1 or type 2 inflammatory pattern. Type 1 inflammation is characterized by the presence of neutrophils and type 1 cytokines, such as interferon-γ (IFN-γ). Type 2 inflammation is characterized by the presence of eosinophils and type 2 cytokines, such as interleukin 4 (IL-4), IL-5 and IL-13. Traditionally, CRSsNP has been associated with type 1 inflammation and CRSwNP with type 2 inflammation. However, eosinophilia has also been shown to be present in CRSsNP [6]. In addition, the traditional type 1 vs. type 2 delineation does not tell the whole story. The roles of IL-17, IL-21, IL-22, IL-26, innate lymphoid cells, and other cellular and soluble mediators are being increasingly recognized in CRS pathophysiology [7,8,9].

Allergy is also characterized by type 2 inflammation and because of this similarity, an association between allergy and CRS (more specifically CRSwNP) has frequently been assumed. However, the mechanisms by which allergy may influence CRS are not inherently obvious. Although allergens enter the nose via inspiration, inspiration alone cannot introduce allergens into the sinuses. A study by Adkins et al. [10] using radiolabeled ragweed pollen and subsequent imaging demonstrated that allergen particles were unable to enter the paranasal sinuses despite being present within the nasal cavity and oropharynx. Furthermore, inflammation and polyposis cause meatal obstruction, which would make direct entry of aeroallergens into the sinuses difficult. 

When allergy plays a significant role in CRS, some evidence points to a systemic process as a potential mechanism. In a sensitized patient, aeroallergens engage nasal dendritic cells, which then activate effector T-helper lymphocytes. Aeroallergens can also be processed by non-professional antigen-presenting cells such as macrophages, B-lymphocytes, mast cells and eosinophils within the nasal cavities to activate allergen-specific effector T lymphocytes [11]. These cells then migrate to the bone marrow. Once in the bone marrow, cytokines associated with allergic inflammation, including IL-4, IL-5 and IL-13, are released which stimulate the production of eosinophils, mast cells and basophils which enter the systemic circulation and recognize adhesion molecules and chemotactic signals. It is via this mechanism that eosinophilia progresses within the nasal cavities following seasonal aeroallergen exposure [12]. However, eosinophils will also be directed into tissues that display relevant adhesion molecules and chemotactic signals. Chronic rhinosinusitis patients express the necessary adhesion molecules and chemotactic machinery to recruit inflammatory cells into the sinuses [13,14], and it is by this mechanism that allergens may exacerbate CRS. A study by Baroody et al. [15] substantiated the concept of a systemic process linking allergy with CRS. Upon performing unilateral nasal allergen challenge, eosinophilia was increased not only in the ipsilateral maxillary sinus but in the contralateral maxillary sinus as well.

In a subset of patients, however, systemic allergy testing is negative but locally-present IgE in the sinonasal cavity may be present, a condition that has been referred to as local allergic rhinitis (LAR) or entopy. The immunological characteristics of LAR are local IgE production, type 2 inflammation and a positive nasal provocation allergen test. Immunoglobin class switching to IgE has been demonstrated in the nasal mucosa, so there is also a plausible mechanism for allergic processes completely confined to the nasal tissues, without systemic involvement [16,17,18].

Overall, these findings demonstrate a potential pathophysiologic link between aeroallergen exposure and CRS. However, further study is necessary to understand the relationship between local and systemic allergic inflammation. Also of note, the majority of the aforementioned studies pertain to patients with eosinophilic chronic rhinosinusitis (with nasal polyposis). The systemic and local mechanisms contributing to other phenotypes, including AFRS and CCAD, need further elucidation.

## 4. Evidence for a Link between Allergic Rhinitis and Chronic Rhinosinusitis: Clinical Evidence

Despite some evidence of pathophysiologic overlap between allergy and CRS, clinical studies have demonstrated contradictory findings. Wilson et al. [5] performed the most comprehensive systematic review evaluating the relationship between allergy and CRS. Twenty-four articles were included in this review. Nearly an equal number of studies supported or refuted an association of allergy with CRS in patients with CRSwNP or CRSsNP.

### 4.1. Chronic Rhinosinusitis with Nasal Polyposis 

CRSwNP is strongly associated with Th2-mediated inflammation [19], as is allergy. In fact, several studies have supported a relationship by demonstrating increased rates of positive skin prick testing (SPT) among CRSwNP patients compared to controls [20,21]. However, several other studies have demonstrated no significant relationship. For example, Erbeck et al. [22] demonstrated no relationship between allergy and polyp size, symptoms or recurrence rate. In the aforementioned review article by Wilson et al. [3], ten studies supported an association between CRSwNP and allergy while seven did not. One study was equivocal. Therefore, despite some overlapping pathophysiologic features, conflicting data exists regarding a relationship between allergy and CRSwNP. 

### 4.2. Chronic Rhinosinusitis without Nasal Polyposis 

Fewer studies have evaluated the relationship between CRSsNP and allergy. Some studies have suggested a higher burden of sinonasal disease in allergic patients based on imaging [23,24]. However, other studies have demonstrated no association [25]. In the article by Wilson et al. [3], four studies supported an association between allergy and CRSsNP and five did not. Again, this demonstrates conflicting data regarding a relationship between allergy and CRSsNP. 

Given these findings, Wilson et al. and subsequently The International Consensus Statement on Allergy and Rhinology: Allergic Rhinitis concluded that the aggregate level of evidence linking allergy to either CRS subtype was level D [4,5]. However, significant limitations exist within the prior literature. First, studies often included patients with and without polyposis in the same cohort [26,27]. Second, even amongst patients with polyposis, certain CRS endotypes may be more closely associated with allergen exposure than others. Yet, it was often not clarified whether these endotypes were included within or excluded from the study cohort, thus potentially skewing the analysis in one direction or the other. For instance, two CRS subtypes that have shown a greater association with allergy are AFRS and the more recently described CCAD, which will both be individually discussed.

## 5. Subtypes of CRS Demonstrating a Link between Allergic Rhinitis and Chronic Rhinosinusitis

### 5.1. Allergic Fungal Rhinosinusitis

Allergic fungal rhinosinusitis (AFRS) is a noninvasive, recurrent subset of CRSwNP that most commonly affects immunocompetent hosts. It is most prevalent within the Mississippi river basin, and southern regions of the United States [28]. In 1994, Bent and Kuhn published five major criteria to establish the diagnosis of AFRS, one of which is a type I hypersensitivity to inhaled fungal elements confirmed by history, skin testing or serology. Therefore by definition, patients with AFRS have allergy. Since then, the role of allergy in AFRS has been reinforced, and sometimes questioned, within the literature. Manning and Holman [29] compared culture-positive *Bipolaris* AFRS patients to non-CRS patients and found *Bipolaris*-specific IgE and IgG antibodies, as well as positive skin prick testing to *Bipolaris* in patients with AFRS. Furthermore, the largest AFRS case series reported from the southwestern United States found that all patients with AFRS had inhalant allergy [30]. However, not all patients with fungal allergy develop AFRS, and conversely not all patients with AFRS demonstrate fungal allergy, raising questions regarding the pathogenesis of this entity [31]. One possible theory to explain the absence of fungal allergy was suggested by Collins et al., who proposed that AFRS might result from a local, rather than a systemic, allergic process [32].

### 5.2. Central Compartment Atopic Disease (CCAD) 

Central compartment atopic disease (CCAD) is a more recently described CRS variant strongly associated with allergy. First described in 2014 [33], although not yet named CCAD, this variant includes polypoid changes of the middle turbinate. In this study, all twenty-five patients tested positive for inhalant allergy. Brunner et al. [34] similarly demonstrated a higher association of allergen sensitization in patients with isolated middle turbinate changes than in those with diffuse polyposis. Later, additional clinical description of this entity, including more advanced forms, was published [35], demonstrating that other central structures including the posterior–superior nasal septum, middle turbinates, and superior turbinates are involved. In this study, 14/15 patients tested positive for inhalant allergies. Hamizan et al. [36] evaluated radiologic findings associated with CCAD, and found that a central pattern of mucosal disease had a higher association with allergy. Overall, this central pattern of inflammatory changes has been shown to have a high association with allergy. Further study is needed to better clarify the etiology and clinical course of this CRS subtype.

## 6. Management

If allergy contributes to CRS, then it would be logical that allergen-targeting therapies would be efficacious in the treatment of CRS. Such treatments include immunotherapy, anti-IgE, and anti-cytokine (IL-4, IL-5, IL-13) therapy, which will be discussed.

### 6.1. Immunotherapy 

Despite a strong recommendation for allergen immunotherapy (IT) in patients with allergic rhinitis (AR), its role in CRS remains less certain. A systematic review by DeYoung et al. [37] looked at sinusitis-specific outcomes in CRS patients who underwent IT. Seven studies were included which demonstrated symptom reduction in the short-term, however the number and quality of studies included deemed this conclusion to be weak. Current CRS treatment recommendations specify allergy testing and treatment as an option.

The role of IT in the treatment of AFRS has also been examined. For instance, Folker et al. [38] demonstrated decreased severity of patient symptoms after receiving IT. Yet overall, studies that have examined the role of allergen-specific immunotherapy in the treatment of AFRS have a relatively low level of evidence. Furthermore, a more recent evidence-based review by Gan et al. [39] concluded, based on a limited number of studies, that there was an equal degree of benefit and harm, and that IT should only be considered as an option for AFRS. The role of immunotherapy in CCAD has not yet been studied.

### 6.2. Anti-Immunoglobulin E Therapy 

Omalizumab is an IgE-targeting therapy, which selectively binds free circulating IgE and decreases the expression of IgE receptors on mast cells, basophils, and dendritic cells. Prior studies have demonstrated its efficacy in the management of recalcitrant CRSwNP [40]. Symptom improvement after treatment with IgE-targeting therapies would provide a compelling argument for the role of allergy in CRS. However, evidence for its efficacy remains mixed and the mechanism of action by which omalizumab is effective in patients with CRSwNP is still being elucidated. Gevaert et al. [41] found significant reduction of polyp size and quality of life improvement with anti-IgE therapy. However, they showed that this improvement was related to local mucosal IgE levels and not total serum IgE level. Therefore, the authors felt that its efficacy may be related to increased local tissue IgE, associated with Staphylococcus aureus enterotoxin, rather than inhalant allergy. Pinto et al. [42] showed non-statistically significant improvements on imaging and Sino-Nasal Outcome Test (SNOT-20) measures after treatment with omalizumab and concluded that IgE only played a small role in the mucosal inflammation of CRS. Therefore, the evidence remains mixed. 

### 6.3. Anti-Cytokine Therapy

Allergy is characterized by a type 2 inflammatory pattern, which includes cytokines IL-4, IL-5 and IL-13. Symptom improvement after treatment with therapies directed at these cytokines would provide additional evidence for the contribution of allergy to CRS. A recent systematic review by Tsetsos et al. [43] evaluated the efficacy of such therapies in patients with CRSwNP. This review included randomized controlled trials, which evaluated anti-IL-5 (reslizumab and mepolizumab) and anti-IL-4/IL-13 (dupilumbab) biologics. Overall, results were encouraging despite the inclusion of few studies with small sample sizes. Reslizumab was shown to reduce nasal polyp size, but only in patients with elevated nasal IL-5 levels [44]. Mepolizumab was found to reduce the need for surgery in CRSwNP patients [45], and dupilumab was found to reduce nasal polyp burden [46].

## 7. Conclusions

Despite an assumed association between allergy and CRS, evidence for a relationship remains debated, especially when patients are subtyped into the broad classification of CRSwNP and CRSsNP. Certain CRS endotypes, however, have demonstrated a stronger association with allergy, including allergic fungal rhinosinusitis (AFRS) and central compartment atopic disease (CCAD). Thus, it is likely that with better-defined CRS categorization, we will be able to better delineate the relationship between allergy and specific CRS endotypes in the future.
